# 1′-Methyl-4′-[4-(trifluoro­meth­yl)phen­yl]dispiro­[indan-2,2′-pyrrolidine-3′,2′′-indan]-1,3,1′′-trione

**DOI:** 10.1107/S1600536811044527

**Published:** 2011-10-29

**Authors:** Ang Chee Wei, Mohamed Ashraf Ali, Tan Soo Choon, Madhukar Hemamalini, Hoong-Kun Fun

**Affiliations:** a Institute for Research in Molecular Medicine, Universiti Sains Malaysia, 11800 USM, Penang, Malaysia; b X-ray Crystallography Unit, School of Physics, Universiti Sains Malaysia, 11800 USM, Penang, Malaysia

## Abstract

In the title compound, C_28_H_20_F_3_NO_3_, the pyrrolidine ring adopts a half-chair conformation. The other five-membered rings adopt envelope conformations with the spiro and methylene C atoms as the flap atoms. In the crystal, mol­ecules are connected *via* weak C—H⋯O hydrogen bonds, forming sheets parallel to the *bc* plane.

## Related literature

For a related structure and background references, see: Wei *et al.* (2011 ▶). For ring puckering parameters, see: Cremer & Pople (1975 ▶). For the stability of the temperature controller used in the data collection, see: Cosier & Glazer (1986 ▶). 
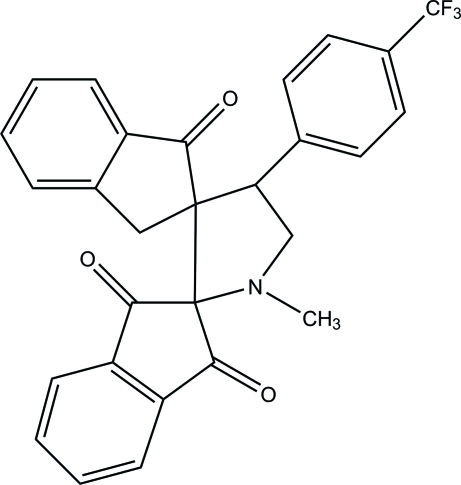


## Experimental

### 

#### Crystal data

C_28_H_20_F_3_NO_3_
*M* *_r_* = 475.45
Monoclinic, 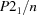

*a* = 7.8070 (2) Å
*b* = 22.0878 (5) Å
*c* = 13.1278 (3) Å
β = 101.420 (2)°
*V* = 2218.93 (9) Å^3^
*Z* = 4
Mo *K*α radiation
μ = 0.11 mm^−1^
*T* = 100 K
0.27 × 0.15 × 0.13 mm

#### Data collection

Bruker SMART APEXII CCD diffractometer
Absorption correction: multi-scan (*SADABS*; Bruker, 2009 ▶) *T*_min_ = 0.971, *T*_max_ = 0.986
22102 measured reflections
6530 independent reflections
3872 reflections with *I* > 2σ(*I*)
*R*_int_ = 0.077

#### Refinement

*R*[*F* ^2^ > 2σ(*F* ^2^)] = 0.070
*wR*(*F* ^2^) = 0.162
*S* = 1.04
6530 reflections
317 parameters
H-atom parameters constrained
Δρ_max_ = 0.48 e Å^−3^
Δρ_min_ = −0.40 e Å^−3^

### 

Data collection: *APEX2* (Bruker, 2009 ▶); cell refinement: *SAINT* (Bruker, 2009 ▶); data reduction: *SAINT*; program(s) used to solve structure: *SHELXTL* (Sheldrick, 2008 ▶); program(s) used to refine structure: *SHELXTL*; molecular graphics: *SHELXTL*; software used to prepare material for publication: *SHELXTL* and *PLATON* (Spek, 2009 ▶).

## Supplementary Material

Crystal structure: contains datablock(s) global, I. DOI: 10.1107/S1600536811044527/hb6459sup1.cif

Structure factors: contains datablock(s) I. DOI: 10.1107/S1600536811044527/hb6459Isup2.hkl

Additional supplementary materials:  crystallographic information; 3D view; checkCIF report

## Figures and Tables

**Table 1:** Hydrogen-bond geometry (Å, °)

*D*—H⋯*A*	*D*—H	H⋯*A*	*D*⋯*A*	*D*—H⋯*A*
C17—H17*A*⋯O3^i^	0.95	2.52	3.130 (3)	122
C23—H23*A*⋯O1^ii^	0.95	2.51	3.104 (3)	121

Symmetry codes: (i) 
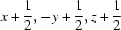
; (ii) 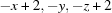.

**Fig. 1.**
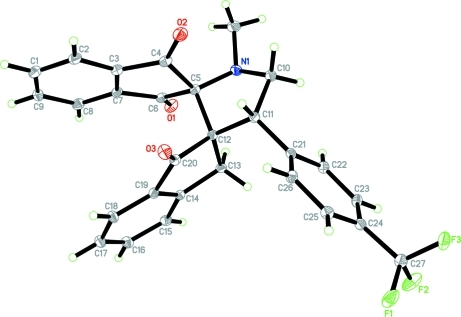
The asymmetric unit of the title compound, showing 30% probability displacement ellipsoids.

**Fig. 2.**
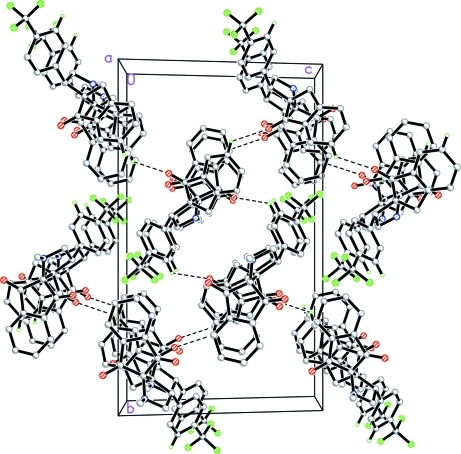
The crystal packing of the title compound (I). H atoms not involved in hydrogen bonding are omitted.

## supplementary crystallographic information

### Comment

As part of our ongoing search for novel heterocyclic compounds with antitubercular activity (Wei *et al.*, 2011), our group has synthesized the title compound as described below.

The asymmetric unit of the title compound is shown in Fig. 1. The pyrrolidine ring (N1/C5/C10–C12) is twisted about the C5 and C12 bonds, with puckering parameters (Cremer & Pople, 1975) Q = 0.457 (2) Å and f = 46.8 (3)°, and adopting a half-chair conformation. The two five- membered carbocyclic rings, C3–C7 and C12–C14/C19,C20, are in envelope conformations: puckering parameters Q = 0.213 (2) Å and f = 76.2 (7)° with atom C5 at the flap; and Q = 0.234 (2) Å and f = 189.1 (6)° with atom C13 at the flap, respectively.  The indane (C1–C9) ring is essentially planar [maximum deviation of  0.208 (2) Å for atom C5] and forms dihedral angles of  28.51 (10)° and 61.94 (9) ° with the terminal phenyl (C14–C19/C21–C26) rings.

In the crystal (Fig. 2), the molecules are connected *via* weak intermolecular C—H···O (Table 1) hydrogen bonds, forming two- dimensional networks parallel to the *bc*-plane.

### Experimental

A mixture of (*E*)2-(4-trifluoromethylbenzylidene)-2,3-dihydro-1*H*- indene-1-one (0.001 mmol), ninhydrin (0.001 mmol) and sarcosine (0.002 mmol) (1:1:2) were dissolved in methanol (10 ml) and refluxed for 4 h. After completion of the reaction as evident from TLC, the mixture was poured into water. The precipitated solid was filtered, washed and recrystallised from petroleum ether-ethyl acetate mixture (1:1) to reveal the title compound as yellow blocks.

### Refinement

All hydrogen atoms were positioned geometrically [ C–H = 0.95–1.00 Å] and were refined using a riding model, with *U*_iso_(H) = 1.2 or 1.5 *U*_eq_(C). A rotating group model was applied to the methyl groups.

### Crystal data

**Table:**

C_28_H_20_F_3_NO_3_	*F*(000) = 984
*M**_r_* = 475.45	*D*_x_ = 1.423 Mg m^−^^3^
Monoclinic, *P*2_1_/*n*	Mo *K*α radiation, λ = 0.71073 Å
Hall symbol: -P 2yn	Cell parameters from 3175 reflections
*a* = 7.8070 (2) Å	θ = 2.4–29.0°
*b* = 22.0878 (5) Å	µ = 0.11 mm^−^^1^
*c* = 13.1278 (3) Å	*T* = 100 K
β = 101.420 (2)°	Block, yellow
*V* = 2218.93 (9) Å^3^	0.27 × 0.15 × 0.13 mm
*Z* = 4	

### Data collection

**Table:**

Bruker SMART APEXII CCD diffractometer	6530 independent reflections
Radiation source: fine-focus sealed tube	3872 reflections with *I* > 2σ(*I*)
graphite	*R*_int_ = 0.077
φ and ω scans	θ_max_ = 30.2°, θ_min_ = 1.8°
Absorption correction: multi-scan (*SADABS*; Bruker, 2009)	*h* = −11→11
*T*_min_ = 0.971, *T*_max_ = 0.986	*k* = −30→31
22102 measured reflections	*l* = −18→18

### Refinement

**Table:**

Refinement on *F*^2^	Primary atom site location: structure-invariant direct methods
Least-squares matrix: full	Secondary atom site location: difference Fourier map
*R*[*F*^2^ > 2σ(*F*^2^)] = 0.070	Hydrogen site location: inferred from neighbouring sites
*wR*(*F*^2^) = 0.162	H-atom parameters constrained
*S* = 1.04	*w* = 1/[σ^2^(*F*_o_^2^) + (0.0624*P*)^2^ + 0.6034*P*] where *P* = (*F*_o_^2^ + 2*F*_c_^2^)/3
6530 reflections	(Δ/σ)_max_ < 0.001
317 parameters	Δρ_max_ = 0.48 e Å^−^^3^
0 restraints	Δρ_min_ = −0.40 e Å^−^^3^

### Special details

**Table:**

Experimental. The crystal was placed in the cold stream of an Oxford Cryosystems Cobra open-flow nitrogen cryostat (Cosier & Glazer, 1986) operating at 100.0 (1) K.
Geometry. All esds (except the esd in the dihedral angle between two l.s. planes) are estimated using the full covariance matrix. The cell esds are taken into account individually in the estimation of esds in distances, angles and torsion angles; correlations between esds in cell parameters are only used when they are defined by crystal symmetry. An approximate (isotropic) treatment of cell esds is used for estimating esds involving l.s. planes.
Refinement. Refinement of F^2^ against ALL reflections. The weighted R-factor wR and goodness of fit S are based on F^2^, conventional R-factors R are based on F, with F set to zero for negative F^2^. The threshold expression of F^2^ > 2sigma(F^2^) is used only for calculating R-factors(gt) etc. and is not relevant to the choice of reflections for refinement. R-factors based on F^2^ are statistically about twice as large as those based on F, and R- factors based on ALL data will be even larger.

### Fractional atomic coordinates and isotropic or equivalent isotropic displacement parameters (Å^2^)

**Table:**

	*x*	*y*	*z*	*U*_iso_*/*U*_eq_	
F1	1.4957 (2)	−0.05331 (7)	0.55766 (14)	0.0444 (4)	
F2	1.4706 (2)	−0.11963 (8)	0.67246 (11)	0.0414 (4)	
F3	1.3006 (2)	−0.12198 (7)	0.52402 (12)	0.0404 (4)	
O1	0.8027 (2)	0.12100 (8)	1.06182 (12)	0.0259 (4)	
O2	0.5579 (2)	0.16217 (8)	0.71690 (12)	0.0283 (4)	
O3	0.9907 (2)	0.18882 (7)	0.73016 (11)	0.0249 (4)	
N1	0.6695 (2)	0.05913 (9)	0.86261 (14)	0.0208 (4)	
C1	0.6883 (3)	0.33200 (12)	0.90164 (19)	0.0264 (5)	
H1A	0.6643	0.3729	0.8813	0.032*	
C2	0.6425 (3)	0.28631 (11)	0.82908 (18)	0.0236 (5)	
H2A	0.5879	0.2952	0.7594	0.028*	
C3	0.6793 (3)	0.22679 (10)	0.86166 (16)	0.0200 (5)	
C4	0.6483 (3)	0.16958 (11)	0.80161 (16)	0.0207 (5)	
C5	0.7496 (3)	0.11868 (10)	0.87093 (16)	0.0172 (5)	
C6	0.7754 (3)	0.14805 (11)	0.97970 (16)	0.0195 (5)	
C7	0.7564 (3)	0.21422 (10)	0.96488 (16)	0.0194 (5)	
C8	0.8017 (3)	0.26022 (11)	1.03752 (17)	0.0228 (5)	
H8A	0.8534	0.2514	1.1077	0.027*	
C9	0.7691 (3)	0.31913 (11)	1.00426 (18)	0.0252 (5)	
H9A	0.8021	0.3515	1.0518	0.030*	
C10	0.7002 (3)	0.03124 (11)	0.76618 (17)	0.0227 (5)	
H10A	0.5973	0.0367	0.7093	0.027*	
H10B	0.7239	−0.0126	0.7763	0.027*	
C11	0.8606 (3)	0.06418 (10)	0.74080 (16)	0.0183 (5)	
H11A	0.8158	0.0925	0.6822	0.022*	
C12	0.9279 (3)	0.10405 (10)	0.83779 (15)	0.0160 (4)	
C13	1.0579 (3)	0.07642 (10)	0.93124 (16)	0.0172 (5)	
H13A	0.9951	0.0585	0.9824	0.021*	
H13B	1.1316	0.0449	0.9078	0.021*	
C14	1.1665 (3)	0.13004 (10)	0.97689 (16)	0.0171 (5)	
C15	1.2772 (3)	0.13615 (11)	1.07356 (16)	0.0209 (5)	
H15A	1.2924	0.1037	1.1221	0.025*	
C16	1.3649 (3)	0.19073 (11)	1.09748 (17)	0.0235 (5)	
H16A	1.4384	0.1957	1.1638	0.028*	
C17	1.3475 (3)	0.23843 (11)	1.02646 (17)	0.0239 (5)	
H17A	1.4090	0.2752	1.0448	0.029*	
C18	1.2409 (3)	0.23229 (10)	0.92931 (17)	0.0210 (5)	
H18A	1.2293	0.2642	0.8798	0.025*	
C19	1.1512 (3)	0.17805 (10)	0.90651 (16)	0.0173 (5)	
C20	1.0218 (3)	0.16199 (10)	0.81240 (16)	0.0178 (5)	
C21	0.9976 (3)	0.02506 (10)	0.70611 (16)	0.0172 (5)	
C22	1.0503 (3)	−0.03063 (10)	0.75231 (16)	0.0195 (5)	
H22A	0.9989	−0.0449	0.8076	0.023*	
C23	1.1761 (3)	−0.06544 (11)	0.71882 (16)	0.0200 (5)	
H23A	1.2106	−0.1032	0.7510	0.024*	
C24	1.2516 (3)	−0.04479 (10)	0.63779 (16)	0.0185 (5)	
C25	1.2032 (3)	0.01078 (10)	0.59215 (16)	0.0203 (5)	
H25A	1.2559	0.0252	0.5375	0.024*	
C26	1.0776 (3)	0.04543 (10)	0.62653 (16)	0.0194 (5)	
H26A	1.0456	0.0836	0.5953	0.023*	
C27	1.3791 (3)	−0.08444 (11)	0.59856 (17)	0.0223 (5)	
C28	0.4895 (3)	0.05533 (12)	0.8773 (2)	0.0291 (6)	
H28A	0.4807	0.0739	0.9439	0.044*	
H28B	0.4542	0.0128	0.8771	0.044*	
H28C	0.4127	0.0768	0.8207	0.044*	

### Atomic displacement parameters (Å^2^)

**Table:**

	*U*^11^	*U*^22^	*U*^33^	*U*^12^	*U*^13^	*U*^23^
F1	0.0479 (11)	0.0266 (9)	0.0723 (12)	0.0028 (7)	0.0446 (9)	0.0036 (8)
F2	0.0411 (10)	0.0529 (11)	0.0326 (8)	0.0268 (8)	0.0128 (7)	0.0114 (7)
F3	0.0405 (10)	0.0351 (9)	0.0443 (9)	0.0066 (7)	0.0053 (7)	−0.0204 (7)
O1	0.0308 (10)	0.0292 (10)	0.0189 (7)	0.0075 (7)	0.0077 (7)	0.0047 (7)
O2	0.0281 (10)	0.0321 (10)	0.0215 (8)	0.0070 (8)	−0.0032 (7)	−0.0025 (7)
O3	0.0298 (10)	0.0234 (9)	0.0200 (8)	−0.0010 (7)	0.0013 (7)	0.0043 (7)
N1	0.0169 (10)	0.0217 (11)	0.0247 (9)	−0.0028 (8)	0.0065 (8)	−0.0023 (8)
C1	0.0215 (12)	0.0231 (13)	0.0346 (13)	0.0046 (10)	0.0054 (10)	0.0021 (10)
C2	0.0190 (12)	0.0252 (13)	0.0259 (11)	0.0034 (10)	0.0028 (9)	0.0038 (10)
C3	0.0174 (11)	0.0214 (13)	0.0212 (10)	0.0023 (9)	0.0036 (9)	0.0008 (9)
C4	0.0182 (11)	0.0258 (13)	0.0187 (10)	0.0036 (9)	0.0048 (8)	0.0022 (9)
C5	0.0163 (11)	0.0180 (12)	0.0178 (9)	0.0022 (9)	0.0043 (8)	−0.0002 (8)
C6	0.0161 (11)	0.0235 (13)	0.0189 (10)	0.0026 (9)	0.0034 (8)	−0.0008 (9)
C7	0.0155 (11)	0.0212 (12)	0.0216 (10)	0.0029 (9)	0.0040 (8)	−0.0007 (9)
C8	0.0221 (12)	0.0259 (13)	0.0202 (10)	0.0034 (10)	0.0037 (9)	−0.0017 (9)
C9	0.0226 (13)	0.0212 (13)	0.0312 (12)	−0.0011 (10)	0.0035 (10)	−0.0072 (10)
C10	0.0180 (12)	0.0239 (13)	0.0276 (11)	−0.0023 (9)	0.0081 (9)	−0.0055 (10)
C11	0.0182 (11)	0.0186 (12)	0.0177 (10)	0.0003 (9)	0.0024 (8)	−0.0012 (9)
C12	0.0164 (11)	0.0153 (11)	0.0162 (9)	0.0001 (8)	0.0028 (8)	0.0000 (8)
C13	0.0145 (11)	0.0178 (12)	0.0190 (10)	0.0018 (8)	0.0023 (8)	0.0013 (8)
C14	0.0161 (11)	0.0162 (12)	0.0196 (10)	0.0012 (9)	0.0050 (8)	−0.0013 (8)
C15	0.0186 (11)	0.0235 (13)	0.0201 (10)	0.0020 (9)	0.0025 (9)	0.0027 (9)
C16	0.0219 (12)	0.0276 (14)	0.0194 (10)	−0.0018 (10)	0.0004 (9)	−0.0047 (9)
C17	0.0232 (12)	0.0212 (13)	0.0280 (11)	−0.0037 (10)	0.0064 (9)	−0.0049 (10)
C18	0.0220 (12)	0.0173 (12)	0.0241 (11)	−0.0027 (9)	0.0059 (9)	0.0005 (9)
C19	0.0178 (11)	0.0163 (12)	0.0182 (10)	0.0007 (8)	0.0049 (8)	−0.0009 (8)
C20	0.0171 (11)	0.0188 (12)	0.0172 (10)	0.0022 (9)	0.0031 (8)	−0.0002 (8)
C21	0.0168 (11)	0.0176 (12)	0.0169 (9)	−0.0030 (8)	0.0022 (8)	−0.0033 (8)
C22	0.0226 (12)	0.0181 (12)	0.0193 (10)	−0.0017 (9)	0.0078 (9)	−0.0001 (9)
C23	0.0248 (12)	0.0160 (12)	0.0195 (10)	0.0011 (9)	0.0051 (9)	0.0006 (8)
C24	0.0184 (11)	0.0188 (12)	0.0185 (10)	−0.0017 (9)	0.0041 (8)	−0.0019 (9)
C25	0.0229 (12)	0.0211 (12)	0.0180 (10)	−0.0020 (9)	0.0064 (9)	0.0022 (9)
C26	0.0220 (12)	0.0164 (12)	0.0195 (10)	0.0017 (9)	0.0034 (9)	0.0014 (8)
C27	0.0259 (13)	0.0201 (13)	0.0221 (10)	−0.0006 (10)	0.0077 (9)	0.0014 (9)
C28	0.0203 (13)	0.0342 (15)	0.0344 (13)	−0.0006 (11)	0.0092 (10)	−0.0032 (11)

### Geometric parameters (Å, °)

**Table:**

F1—C27	1.336 (3)	C12—C20	1.544 (3)
F2—C27	1.335 (3)	C12—C13	1.554 (3)
F3—C27	1.335 (3)	C13—C14	1.510 (3)
O1—C6	1.214 (3)	C13—H13A	0.9900
O2—C4	1.204 (2)	C13—H13B	0.9900
O3—C20	1.213 (2)	C14—C15	1.393 (3)
N1—C5	1.451 (3)	C14—C19	1.396 (3)
N1—C28	1.458 (3)	C15—C16	1.391 (3)
N1—C10	1.470 (3)	C15—H15A	0.9500
C1—C2	1.385 (3)	C16—C17	1.396 (3)
C1—C9	1.399 (3)	C16—H16A	0.9500
C1—H1A	0.9500	C17—C18	1.385 (3)
C2—C3	1.395 (3)	C17—H17A	0.9500
C2—H2A	0.9500	C18—C19	1.390 (3)
C3—C7	1.397 (3)	C18—H18A	0.9500
C3—C4	1.484 (3)	C19—C20	1.476 (3)
C4—C5	1.560 (3)	C21—C26	1.393 (3)
C5—C6	1.545 (3)	C21—C22	1.397 (3)
C5—C12	1.572 (3)	C22—C23	1.386 (3)
C6—C7	1.478 (3)	C22—H22A	0.9500
C7—C8	1.390 (3)	C23—C24	1.391 (3)
C8—C9	1.380 (3)	C23—H23A	0.9500
C8—H8A	0.9500	C24—C25	1.385 (3)
C9—H9A	0.9500	C24—C27	1.492 (3)
C10—C11	1.541 (3)	C25—C26	1.388 (3)
C10—H10A	0.9900	C25—H25A	0.9500
C10—H10B	0.9900	C26—H26A	0.9500
C11—C21	1.513 (3)	C28—H28A	0.9800
C11—C12	1.552 (3)	C28—H28B	0.9800
C11—H11A	1.0000	C28—H28C	0.9800
			
C5—N1—C28	117.03 (19)	C14—C13—H13B	111.1
C5—N1—C10	107.67 (18)	C12—C13—H13B	111.1
C28—N1—C10	114.55 (18)	H13A—C13—H13B	109.0
C2—C1—C9	121.3 (2)	C15—C14—C19	119.1 (2)
C2—C1—H1A	119.3	C15—C14—C13	129.5 (2)
C9—C1—H1A	119.3	C19—C14—C13	111.44 (17)
C1—C2—C3	117.8 (2)	C16—C15—C14	118.6 (2)
C1—C2—H2A	121.1	C16—C15—H15A	120.7
C3—C2—H2A	121.1	C14—C15—H15A	120.7
C2—C3—C7	120.5 (2)	C15—C16—C17	121.7 (2)
C2—C3—C4	129.64 (19)	C15—C16—H16A	119.2
C7—C3—C4	109.86 (19)	C17—C16—H16A	119.2
O2—C4—C3	127.6 (2)	C18—C17—C16	120.2 (2)
O2—C4—C5	125.3 (2)	C18—C17—H17A	119.9
C3—C4—C5	107.07 (17)	C16—C17—H17A	119.9
N1—C5—C6	115.19 (18)	C17—C18—C19	117.9 (2)
N1—C5—C4	116.63 (18)	C17—C18—H18A	121.1
C6—C5—C4	101.22 (17)	C19—C18—H18A	121.1
N1—C5—C12	100.66 (17)	C18—C19—C14	122.61 (19)
C6—C5—C12	112.44 (17)	C18—C19—C20	128.3 (2)
C4—C5—C12	111.17 (17)	C14—C19—C20	109.03 (19)
O1—C6—C7	126.8 (2)	O3—C20—C19	127.6 (2)
O1—C6—C5	125.6 (2)	O3—C20—C12	125.24 (19)
C7—C6—C5	107.60 (17)	C19—C20—C12	107.10 (17)
C8—C7—C3	121.5 (2)	C26—C21—C22	118.2 (2)
C8—C7—C6	128.83 (19)	C26—C21—C11	119.3 (2)
C3—C7—C6	109.68 (19)	C22—C21—C11	122.5 (2)
C9—C8—C7	117.8 (2)	C23—C22—C21	121.1 (2)
C9—C8—H8A	121.1	C23—C22—H22A	119.4
C7—C8—H8A	121.1	C21—C22—H22A	119.4
C8—C9—C1	121.1 (2)	C22—C23—C24	119.7 (2)
C8—C9—H9A	119.5	C22—C23—H23A	120.2
C1—C9—H9A	119.5	C24—C23—H23A	120.2
N1—C10—C11	105.46 (18)	C25—C24—C23	120.1 (2)
N1—C10—H10A	110.6	C25—C24—C27	121.0 (2)
C11—C10—H10A	110.6	C23—C24—C27	119.0 (2)
N1—C10—H10B	110.6	C24—C25—C26	119.9 (2)
C11—C10—H10B	110.6	C24—C25—H25A	120.1
H10A—C10—H10B	108.8	C26—C25—H25A	120.1
C21—C11—C10	116.65 (19)	C25—C26—C21	121.1 (2)
C21—C11—C12	115.24 (17)	C25—C26—H26A	119.5
C10—C11—C12	104.37 (17)	C21—C26—H26A	119.5
C21—C11—H11A	106.6	F2—C27—F3	105.84 (19)
C10—C11—H11A	106.6	F2—C27—F1	106.4 (2)
C12—C11—H11A	106.6	F3—C27—F1	106.00 (19)
C20—C12—C11	113.04 (17)	F2—C27—C24	112.87 (18)
C20—C12—C13	103.42 (16)	F3—C27—C24	112.13 (19)
C11—C12—C13	119.14 (18)	F1—C27—C24	113.0 (2)
C20—C12—C5	111.96 (18)	N1—C28—H28A	109.5
C11—C12—C5	99.75 (16)	N1—C28—H28B	109.5
C13—C12—C5	109.75 (17)	H28A—C28—H28B	109.5
C14—C13—C12	103.47 (17)	N1—C28—H28C	109.5
C14—C13—H13A	111.1	H28A—C28—H28C	109.5
C12—C13—H13A	111.1	H28B—C28—H28C	109.5
			
C9—C1—C2—C3	0.2 (4)	C6—C5—C12—C11	168.65 (18)
C1—C2—C3—C7	1.3 (4)	C4—C5—C12—C11	−78.7 (2)
C1—C2—C3—C4	−178.9 (2)	N1—C5—C12—C13	−80.37 (19)
C2—C3—C4—O2	−14.0 (4)	C6—C5—C12—C13	42.7 (2)
C7—C3—C4—O2	165.8 (2)	C4—C5—C12—C13	155.43 (17)
C2—C3—C4—C5	168.4 (2)	C20—C12—C13—C14	22.8 (2)
C7—C3—C4—C5	−11.9 (3)	C11—C12—C13—C14	149.22 (19)
C28—N1—C5—C6	65.2 (2)	C5—C12—C13—C14	−96.8 (2)
C10—N1—C5—C6	−164.16 (18)	C12—C13—C14—C15	164.5 (2)
C28—N1—C5—C4	−53.3 (3)	C12—C13—C14—C19	−17.6 (2)
C10—N1—C5—C4	77.4 (2)	C19—C14—C15—C16	1.8 (3)
C28—N1—C5—C12	−173.66 (17)	C13—C14—C15—C16	179.6 (2)
C10—N1—C5—C12	−43.0 (2)	C14—C15—C16—C17	−1.5 (4)
O2—C4—C5—N1	−32.4 (3)	C15—C16—C17—C18	0.1 (4)
C3—C4—C5—N1	145.4 (2)	C16—C17—C18—C19	1.0 (4)
O2—C4—C5—C6	−158.2 (2)	C17—C18—C19—C14	−0.7 (4)
C3—C4—C5—C6	19.6 (2)	C17—C18—C19—C20	175.5 (2)
O2—C4—C5—C12	82.2 (3)	C15—C14—C19—C18	−0.7 (3)
C3—C4—C5—C12	−100.1 (2)	C13—C14—C19—C18	−178.9 (2)
N1—C5—C6—O1	31.2 (3)	C15—C14—C19—C20	−177.5 (2)
C4—C5—C6—O1	158.0 (2)	C13—C14—C19—C20	4.3 (3)
C12—C5—C6—O1	−83.3 (3)	C18—C19—C20—O3	13.6 (4)
N1—C5—C6—C7	−147.48 (19)	C14—C19—C20—O3	−169.8 (2)
C4—C5—C6—C7	−20.7 (2)	C18—C19—C20—C12	−165.5 (2)
C12—C5—C6—C7	98.0 (2)	C14—C19—C20—C12	11.1 (2)
C2—C3—C7—C8	−1.2 (4)	C11—C12—C20—O3	29.4 (3)
C4—C3—C7—C8	179.0 (2)	C13—C12—C20—O3	159.7 (2)
C2—C3—C7—C6	178.0 (2)	C5—C12—C20—O3	−82.3 (3)
C4—C3—C7—C6	−1.9 (3)	C11—C12—C20—C19	−151.44 (18)
O1—C6—C7—C8	15.4 (4)	C13—C12—C20—C19	−21.2 (2)
C5—C6—C7—C8	−165.9 (2)	C5—C12—C20—C19	96.9 (2)
O1—C6—C7—C3	−163.7 (2)	C10—C11—C21—C26	140.2 (2)
C5—C6—C7—C3	15.0 (2)	C12—C11—C21—C26	−96.9 (2)
C3—C7—C8—C9	−0.4 (4)	C10—C11—C21—C22	−41.1 (3)
C6—C7—C8—C9	−179.4 (2)	C12—C11—C21—C22	81.9 (3)
C7—C8—C9—C1	1.9 (4)	C26—C21—C22—C23	−1.3 (3)
C2—C1—C9—C8	−1.8 (4)	C11—C21—C22—C23	−180.0 (2)
C5—N1—C10—C11	22.4 (2)	C21—C22—C23—C24	0.0 (3)
C28—N1—C10—C11	154.43 (19)	C22—C23—C24—C25	1.1 (3)
N1—C10—C11—C21	136.47 (19)	C22—C23—C24—C27	−176.4 (2)
N1—C10—C11—C12	8.1 (2)	C23—C24—C25—C26	−0.9 (3)
C21—C11—C12—C20	79.4 (2)	C27—C24—C25—C26	176.6 (2)
C10—C11—C12—C20	−151.33 (18)	C24—C25—C26—C21	−0.5 (3)
C21—C11—C12—C13	−42.3 (3)	C22—C21—C26—C25	1.5 (3)
C10—C11—C12—C13	86.9 (2)	C11—C21—C26—C25	−179.70 (19)
C21—C11—C12—C5	−161.54 (18)	C25—C24—C27—F2	151.1 (2)
C10—C11—C12—C5	−32.3 (2)	C23—C24—C27—F2	−31.4 (3)
N1—C5—C12—C20	165.38 (16)	C25—C24—C27—F3	−89.5 (3)
C6—C5—C12—C20	−71.5 (2)	C23—C24—C27—F3	88.0 (2)
C4—C5—C12—C20	41.2 (2)	C25—C24—C27—F1	30.2 (3)
N1—C5—C12—C11	45.54 (19)	C23—C24—C27—F1	−152.2 (2)

### Hydrogen-bond geometry (Å, °)

**Table:**

*D*—H···*A*	*D*—H	H···*A*	*D*···*A*	*D*—H···*A*
C17—H17A···O3^i^	0.95	2.52	3.130 (3)	122
C23—H23A···O1^ii^	0.95	2.51	3.104 (3)	121

Symmetry codes: (i) *x*+1/2, −*y*+1/2, *z*+1/2; (ii) −*x*+2, −*y*, −*z*+2.
